# *Calligonum caput-medusae* seedlings adapt to drought stress through changing chlorophyll fluorescence parameters

**DOI:** 10.3389/fpls.2025.1640412

**Published:** 2025-08-07

**Authors:** Haixia Huo, Muhammad Tauseef Jaffar, Jianguo Zhang, Jianxuan Shang

**Affiliations:** ^1^ College of Hydraulic Engineering, Shaanxi A&F Technology University, Yangling, China; ^2^ College of Natural Resources and Environment, Northwest A&F University, Yangling, China; ^3^ National Key Laboratory of Clean Coal Grading Conservation, Shaaxi Coal and Chemical Industry Group Co., Xi’an, China

**Keywords:** *Calligonum caput-medusae*, drought stress, photosystem II, physiology, soil moisture

## Abstract

Understanding plants responses to drought stress is crucial for selecting appropriate species for shelter-forest construction in arid and semi-arid regions. *Calligonum caput-medusae*, one of the most planted shrubs along the Taklimakan Desert Highway Shelterbelt (TDHS), contributes significantly to maintaining the highway’s ecological stability. This study aimed to investigate the physiological responses of biennial *C. caput-medusae* seedlings to drought stress by monitoring changes in soil moisture and chlorophyll fluorescence parameters [actual photo chemical efficiency of PSII (Y_(II)_), unregulated energy dissipation quantum yield (Y_(NO)_), non-photochemical quenching coefficient (NPQ), and regulatory energy dissipation quantum yield (Y_(NPQ))_] under controlled conditions. The results showed that soil moisture declined progressively with prolonged drought stress. Although the photosystem II (PSII) reaction centers of the seedlings experienced some stress after 30 days of drought, no irreversible photodamage occurred. However, the risk of photoinhibition and damage to the photosynthetic apparatus increased with prolonged drought, as evidenced by an increase in NPQ. These findings suggest that *C. caput-medusae* seedlings adapt to drought stress by modulating their chlorophyll fluorescence characteristics, enhancing our understanding of its drought adaptation mechanisms and highlighting the need for future research on its long-term physiological responses under field conditions and varying drought intensities.

## 1 Introduction

Drought is one of the most common limiting factors for plant growth, particularly in arid regions, and plants have evolved various ecological strategies to survive under such harsh conditions (Li C.S. et al., 2022; Li C.J. et al., 2022; Long et al., 2024; Wang et al., 2024). Changes in chlorophyll fluorescence parameters are closely associated with multiple processes involved in photosynthesis (Wittmann and Pfanz, 2016; Jumrani et al., 2017). The effects of environmental stress on photosynthesis can be revealed by the dynamic changes in intrinsic chlorophyll fluorescence, which also reflect internal physiological alterations in plants (Ren et al., 2017). Under normal growth conditions, chlorophyll fluorescence primarily originates from chlorophyll a in photosystem II (PSII), and variations in photosynthetic performance can be effectively monitored through fluorescence signals (Ogawa et al., 2017). Notably, PSII reaction centers are highly sensitive to environmental stress (Ivanov et al., 2008). Therefore, the effects of biotic and abiotic stresses can be assessed by analyzing the kinetic changes in chlorophyll fluorescence parameters in plant leaves (Han et al., 2010; Krasnovsky and Kovalev, 2014). Under stress conditions such as drought, salinity, heavy metal exposure, intense light, or extreme temperatures, alterations in chlorophyll fluorescence parameters provide insight into the structural and functional changes within the photosynthetic apparatus (Hussain et al., 2019). Yao et al. (2018) demonstrated that combining dynamic chlorophyll fluorescence analysis with fluorescence imaging technology offers a comprehensive assessment of both photosynthetic performance and secondary metabolism under drought stress, enabling early detection of plant responses. Sommer et al. (2024) showed that high temperature stress significantly reduced the maximum photochemical efficiency and electron transfer rate of wheat leaves. Therefore, investigating photosynthetic responses and physiological changes of plants under stress environment is essential for understanding plant adaptive mechanisms to environmental stressors.

The Taklimakan Desert Highway (TDH) is the longest highway in the world that continuously traverses a shifting desert. However, frequent and intense wind-sand activity poses significant challenges to its normal operation. Since 2005, the shelterbelt established along the TDH has played a crucial role in maintaining its functionality. The tree and shrub species (such as *Calligonum* spp., *Tamarix* spp., and *Haloxylon ammodendron*), used in the shelterbelt exhibit strong resistance to drought and salinity, and provide essential windbreak and sand-fixation functions (Li et al., 2015, Zhang et al., 2016a). *Calligonum* L. belongs to the Polygonaceae family and recognized as a pioneer tree species for wind and sand prevention in many desert areas of China. These plants are characterized by their strong drought tolerance, resistance to wind erosion, and rapid growth (Xu et al., 2008). They play an important role in windbreak and sand fixation, soil and water conservation, and the maintenance of ecological stability in desert ecosystems (Dounavi et al., 2016; Zhang et al., 2019; Liu et al., 2024).

Previous studies on *Calligonum* species have primarily focused on general physiological traits (Li C.J. et al., 2022), gas exchange parameters (Yan et al., 2010), and seed germination (Tao and Ren, 2004), but detailed insights into their drought adaptation mechanisms remain scarce. This study addresses this research gap by investigating the dynamic responses of *C. caput-medusae* seedlings to drought stress, with a focus on chlorophyll fluorescence parameters and associated physiology. We hypothesized that chlorophyll fluorescence parameters would change systematically under progressive drought stress, indicating adaptive mechanisms of *C. caput-medusae* seedlings. Our objectives were to (i) clarify the adaptive strategies of *C. caput-medusae* under drought conditions, and (ii) determine the drought threshold at which the seedlings exhibit signs of physiological dormancy. These findings aim to contribute novel insights into plant drought tolerance in desert ecosystems and offer a scientific basis for the sustainable management of shelterbelt vegetation along the TDHS.

## 2 Materials and methods

### 2.1 Study area

The study was carried out in 2024 at the Taklimakan Desert Research Station of Chinese Academy of Sciences, located in the hinterland of Taklimakan Desert, Xinjiang, P.R. China (38° 68’ N, 83° 37’ E, 1093 m.a.s.l.). The region is characterized by an extremely arid climate and frequent wind-blown sand activity. The average annual temperature is 12.4 °C, with the coldest average monthly temperature of –8.1 °C in December and the hottest of 28.2 °C in July (He et al., 2002). Annual precipitation averages only 23.7 mm, occurring primarily between May and September, while potential annual evaporation reaches as high as 3,639 mm (Zhang et al., 2016b; Ma et al., 2022).

The soil type is mainly dominated by shifting aeolian sandy soil, which has a very weak pedogenic development. Basic soil physicochemical properties are shown in **Table 1**. The shelterbelt vegetation consists mainly of drought- and salt-tolerant shrub species with strong windbreak and sand fixation capabilities, including *Tamarix* spp., *Haloxylon ammodendron*, and *Calligonum* spp. Due to the absence of surface water resources along the TDH, all plants in the shelterbelt are irrigated via drip systems using local saline groundwater, which has a salinity ranging from 2.8 to 30.0 g L^-^¹ (Zhang et al., 2016a).

**Table 1:** Physicochemical properties of the soil in the study area.

pH (1:5)	EC (dS m^–1^)	Total salt content (g kg^–1^)	Bulk density (g cm^–3^)	Particle composition (%)			
				Clay (<0.002 mm)	Silt (0.002–0.02 mm)	Fine sand (0.02–0.2 mm)	Coarse sand (0.2–2 mm)
8.26	0.437	1.31	1.49	0.27	12.35	82.83	4.54

### 2.2 Experiment design

**Figure 1:**
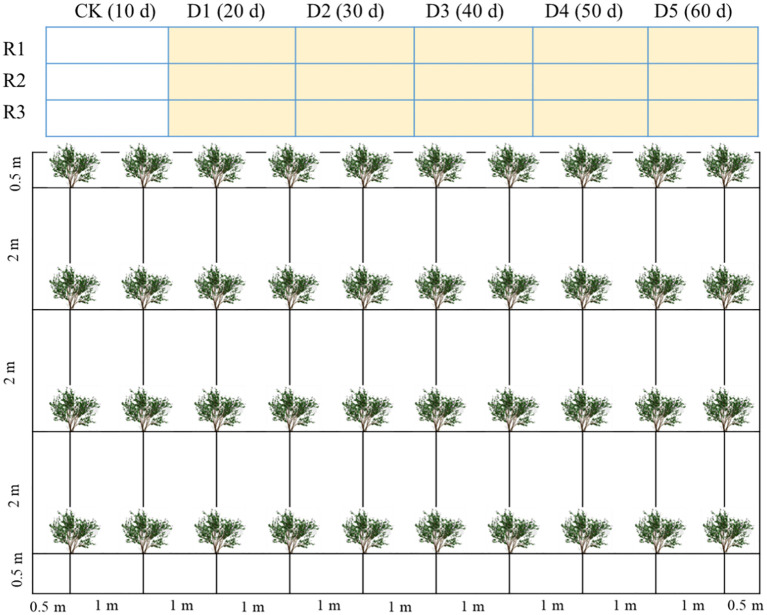
The layout of experimental plots and plant arrangement within each plot. D1, D2, D3, D4, and D5: drought stress treatments, CK is the control. Rl, R2, and R3 are replicates.

The experiment was conducted from July 7 to October 16, 2024. Prior to the onset of drought stress, uniform biennial *C. caput-medusae* seedlings were planted in mid-April across 18 plots (7 m × 10 m each) with a row spacing of 1 m × 2 m. The seedlings were regularly irrigated using local saline groundwater (4.8 g L^-^¹) through drip irrigation, applied once every 10 days at a rate of 25–30 L per plant. The layout of the experimental plots and the arrangement of the plants are illustrated in **Figure 1**. To prevent water movement between plots, impermeable membranes were installed between them prior to planting.

The drought stress experiment consisted of six treatments across a total of 18 plots, with three replicates per treatment. Prior to the initiation of drought stress, all plots were regularly drip-irrigated with saline water (4.8 g L^-^¹). Drought stress was applied to treatments 2–6, while treatment 1 (CK) remained well-irrigated. To simulate varying durations of drought stress, each drought-treated group was re-irrigated sequentially at 10-day intervals. Specifically, treatment D1 was re-irrigated on day 20 (after 10 days of drought), D2 on day 30 (after 20 days of drought), D3 on day 40 (after 30 days of drought), D4 on day 50 (after 40 days of drought), and D5 on day 60 (after 50 days of drought). The control treatment (CK) was re-irrigated on day 10 and did not experience any drought stress. Throughout the entire drought stress period, physiological indices of *C. caput-medusae* seedlings were monitored.

### 2.3 Soil sampling and analysis

Soil samples were collected from beneath the *C. caput-medusae* seedlings at the time of physiological index measurement. Given that the root system of *C. caput-medusae* is primarily distributed within the top 1 m of soil (Wang et al., 2008), samples were taken at 10 cm intervals along the 0–100 cm soil profile, with three replicates per treatment, approximately 30 g was used to determine soil water content using the gravimetric method (oven-dried at 105 °C for 12 hours).

### 2.4 Determination of environmental indicators, plant physiological indicators and litter

During the drought stress experiment, various environmental and plant physiological parameters were measured using a multifunctional plant analysis system (PhotosynQ, USA). These included atmospheric humidity, atmospheric temperature, leaf temperature, photosynthetically active radiation (PAR), linear electron flow (LEF), non-photochemical quenching coefficient (NPQ), actual photochemical efficiency (Y_(II)_), regulatory energy dissipation (Y_(NPQ)_) and non-regulatory energy dissipation (Y_(NO)_). In addition, seedling height and crown width were recorded before and after the drought stress period using a steel measuring tape with 1.0 mm accuracy, while ground diameters of the seedlings were determined with a digital vernier caliper. At the end of experiments, the litters under different treatments were collected, oven-dried at 70–80 °C for 48 hours and weighed using an electronic balance. It is important to note that for treatment D5, the seedlings had died by day 60 and no leaves remained; therefore, data for environmental and plant physiological indicators were not available for this treatment.

### 2.5 Data analysis

The statistical analyses were conducted using SPSS 20.0 (IBM, USA). One-way ANOVA was employed to assess the effects of treatments, followed by Tukey’s test to determine significant differences among means at *p<0.05*. Graphical illustrations were generated with Origin 2021 (OriginLab, USA).

## 3 Results

### 3.1 Temporal variation in soil moisture

**Figure 2:**
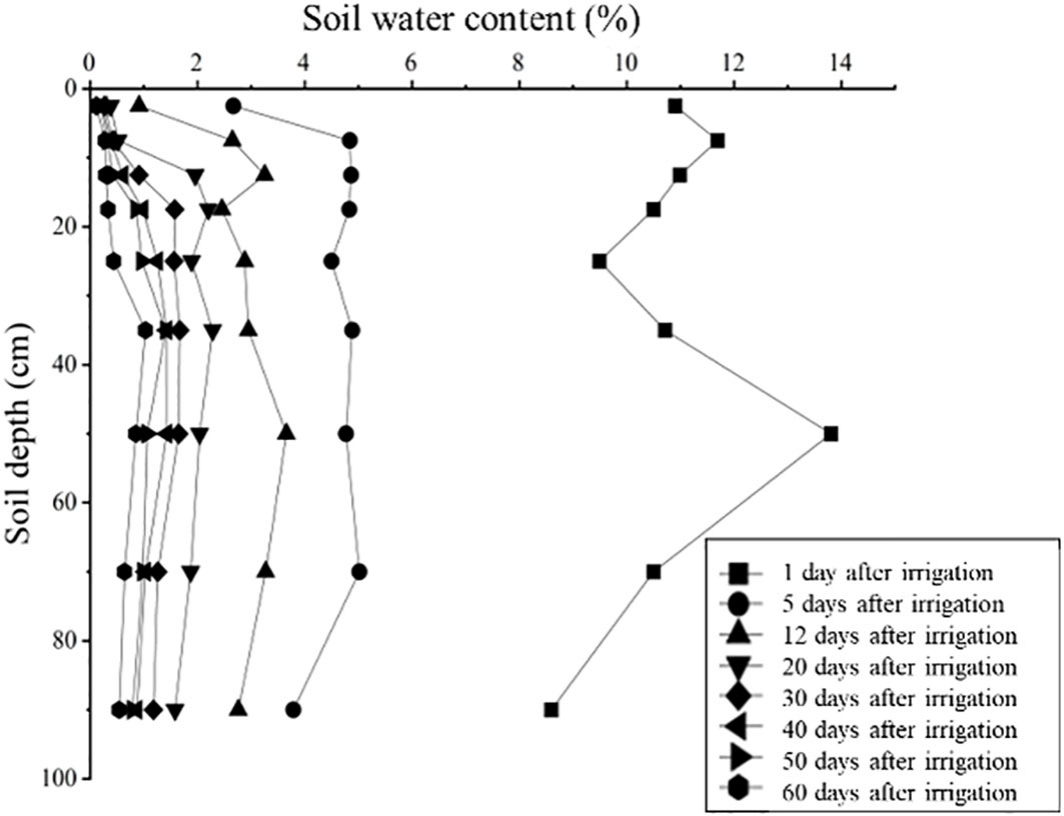
The dynamics of soil water content under continuous drought stress.

As shown in **Figure 2**, the highest soil water content (13.80%) was observed in the 40–60 cm layer one day after irrigation, followed by a sharp decline across all soil layers from 0 to 100 cm within 12 days after drip irrigation. After 20 days of drought stress, soil water content in different soil layers showed a unimodal distribution, with the highest value recorded at the 30–40 cm layer. Following irrigation for 20, 30, 40, 50 and 60 days, the average soil moisture in the 0–100 cm soil layers continued to decrease, reaching 1.64%, 1.17%, 0.91%, 0.78% and 0.50%, respectively.

### 3.2 Diurnal patterns of environmental variables

**Figure 3:**
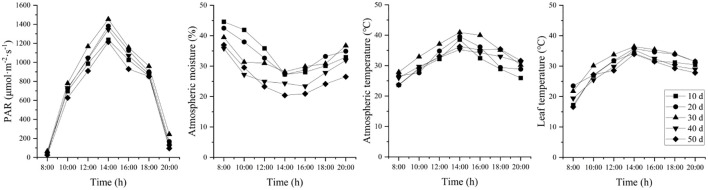
Changes in photosynthetically active radiation (PAR), atmospheric moisture, atmospheric temperature, and leaf temperature during drought stress.

Under different stages of drought stress, environmental factors such as PAR, atmospheric humidity, air temperature and leaf temperature showed consistent diurnal patterns (**Figure 3**). No data was recorded for 60 days due to complete leaf loss on the seedlings. PAR followed a unimodal diurnal curve, peaking at 14:00 with the highest intensity. Atmospheric humidity showed a “V”-shaped pattern, decreasing sharply from 08:00 to 14:00 and then gradually increasing. Both air temperature and leaf temperature increased from 08:00 to 14:00 and subsequently decreased until 20:00. The maximum air and leaf temperatures were 40.92 °C and 36.42 °C, respectively, recorded at 14:00.

### 3.3 Litter accumulation over time

**Figure 4:**
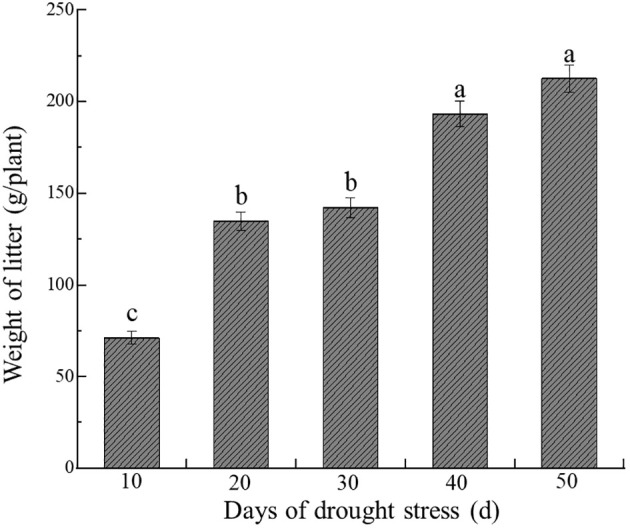
Dynamics of *C. caput-medusae* seedling litter mass under drought stress. The different lowercase letters mean the differences among different treatments. (*p*<0.05).

Compared with CK, different drought stress treatments significantly increased the litter production of *C. caput-medusae* seedlings, with litter amounts rising progressively with prolonged drought stress (*p*<0.05, **Figure 4**). After 20, 30, 40 and 50 days of drought stress, the litter increased by 89.32%, 99.96%, 171.81% and 198.89%, respectively, compared to CK. This suggests that the seedlings adapted to drought stress by shedding fresh and tender assimilation twigs to minimize water and energy loss.

### 3.4 Temporal changes in chlorophyll fluorescence parameters

#### 3.4.1 Dynamics of LEF

**Figure 5:**
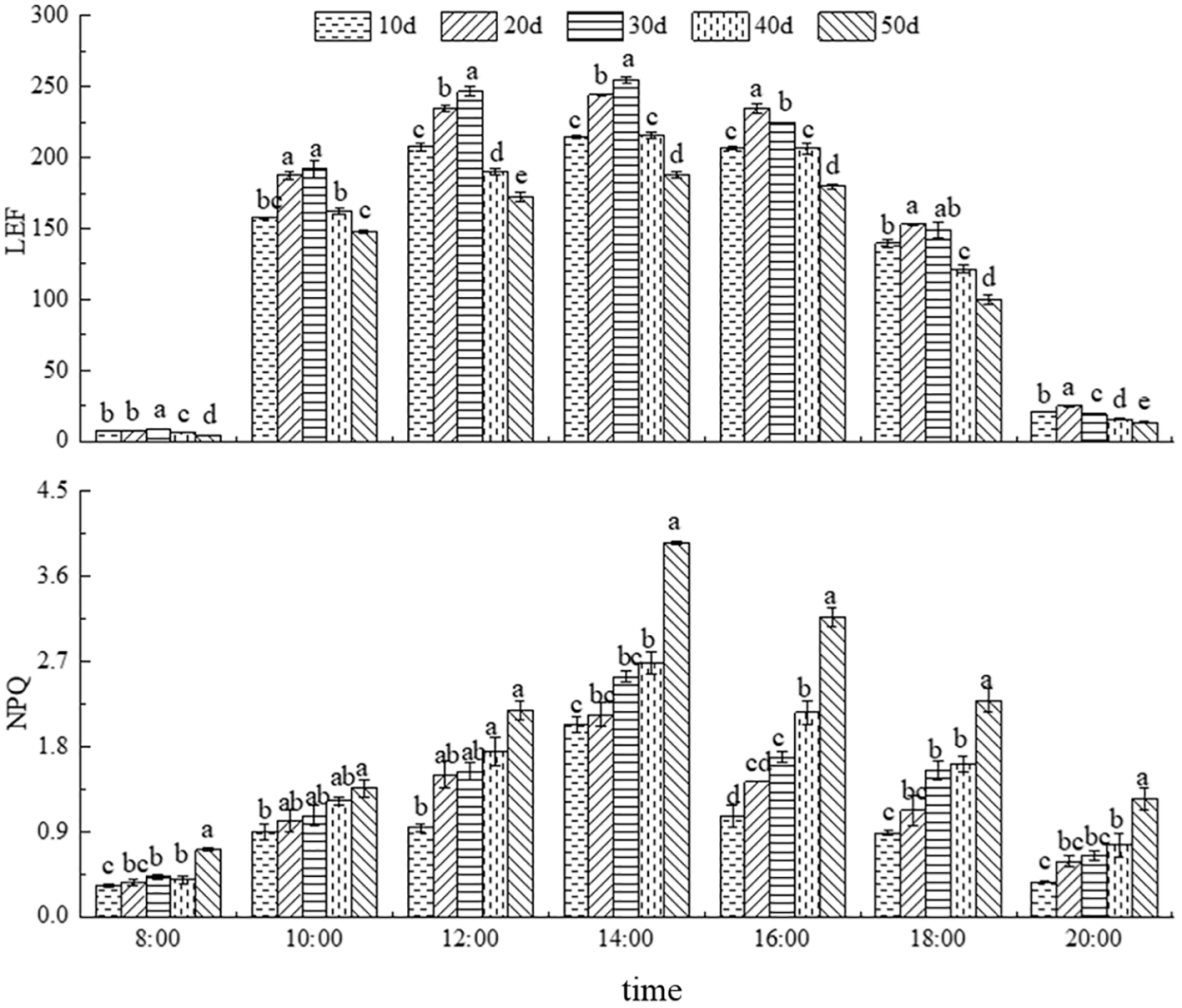
Daily dynamics of linear electron transport (LEF) and non-photochemical quenching coefficient (NPQ) under different drought stress treatments. Different lowercase letters denote the difference of fluorescence parameters in seedlings under different drought stress at the same time (*p*<0.05).

As shown in **Figure 5**, the diurnal variation of LEF in *C. caput-medusae* seedlings initially increased and then decreased under different drought stress treatments, peak values occurring around 14:00. The LEF values after 20 and 30 days of drought stress were higher than those of the CK (10 days), whereas LEF was consistently lower throughout the day after 40 and 50 days of drought stress. According to **Table 2**, the maximum LEF was observed after 30 days of drought stress. The daily mean LEF was significantly higher under 20- and 30-days drought stress but significantly lower under 50 days’ drought stress compared to normal irrigation (10 days). The results demonstrated that *C. caput-medusae* seedlings adapt to moderate drought stress through increasing LEF, while under severe drought stress, they reduce LEF as a protective response.

**Table 2:** Analysis of variance of daily chlorophyll fluorescence parameters (LEF, NPQ, Y_(II)_, Y_(NPQ)_, and Y_(NO)_) of the *C. caput-medusae* seedlings under different drought stress periods.

Drought stress period (d)	LEF	NPQ	Y_(II)_	Y_(NPQ)_	Y_(NO)_
10	136.23b	0.93e	0.58a	0.23d	0.19a
20	155.25a	1.17d	0.50b	0.34c	0.16ab
30	156.26a	1.35c	0.48b	0.35c	0.17a
40	131.01b	1.51b	0.43c	0.40b	0.16ab
50	115.04c	2.15a	0.40c	0.47a	0.13b

Different lowercase letters indicate significant differences among treatments for the same index (*p <*0.05).

#### 3.4.2 Dynamics of NPQ

The diurnal variation in NPQ under different drought stress treatments showed an initial increase followed by a decrease, with peak values occurring around 14:00 (**Figure 5**). NPQ increased with the extension of drought stress. This indicates that, as drought stress intensifies, *C. caput-medusae* seedlings enhance heat dissipation to safely dissipate excess light energy, thereby adapting to the drought environment.

#### 3.4.3 Dynamics of Y_(II)_, Y_(NPQ)_ and Y_(NO)_

**Figure 6:**
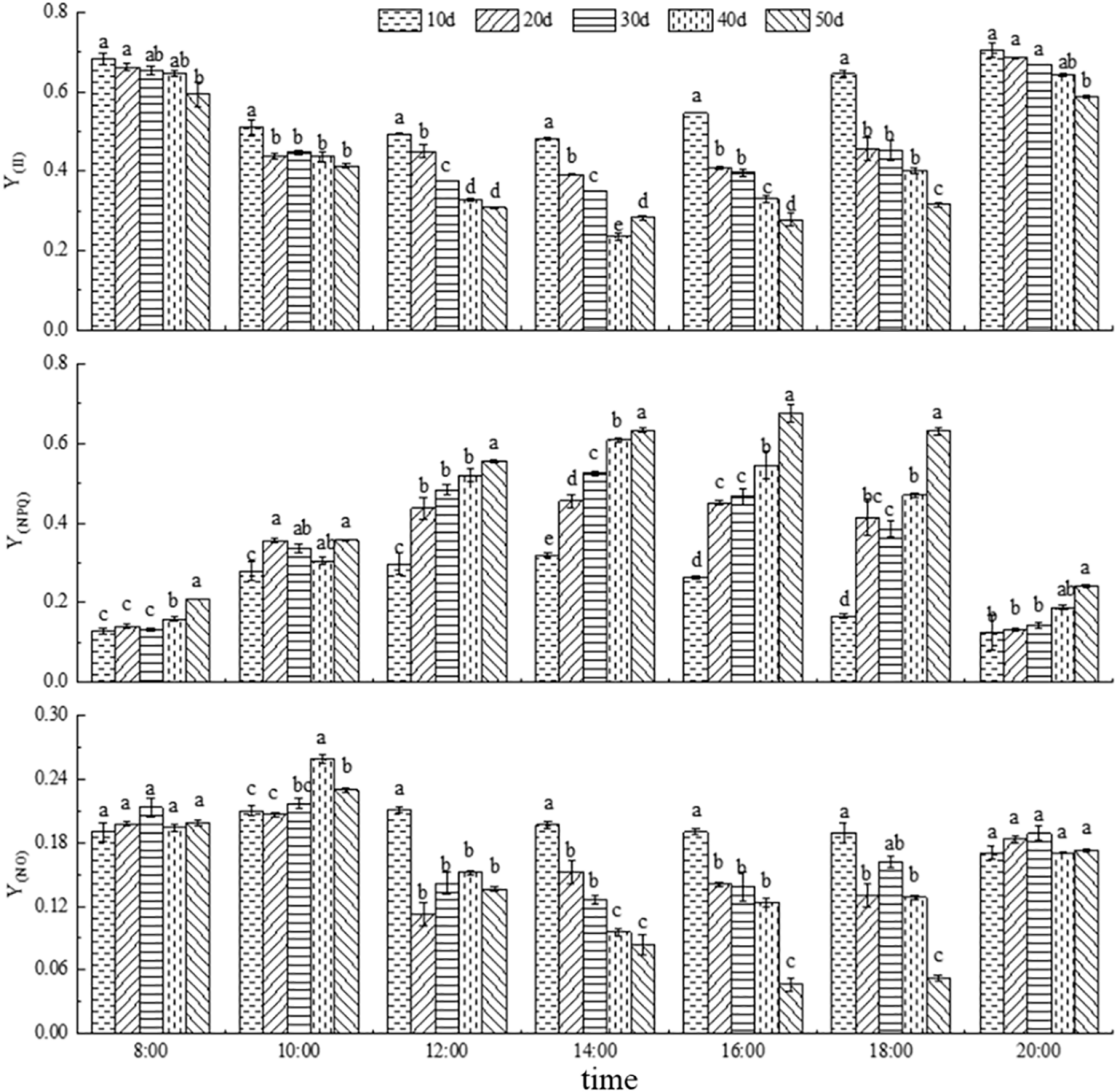
Light energy changes in photosynthetic fluorescence parameters (Y_(II)_, Y_(NPQ)_, and Y_(NO)_) under drought stress. Different lowercase letters denote the difference of fluorescence parameters under different drought stress at the same time (*p*<0.05).

As shown in **Figure 6**, with the progression of drought stress, Y_(II)_ gradually decreased, whereas Y_(NPQ)_ gradually increased, and Y_(NO)_ initially increased and then decreased. The diurnal variation of Y_(II)_ showed a typical decrease followed by an increase, with the lowest values occurring around 14:00. Overall, the Y_(II)_ after 50 days of drought stress was significantly lower than that under the other treatments. In contrast, Y_(NPQ)_ exhibited a diurnal pattern of increase followed by decrease, peaking between 14:00 and 16:00, and its values under 50-day drought stress were significantly higher than those observed in most other treatments.

Y_(NO)_ showed a decreasing trend with the prolongation of drought stress and ranged between 0.05 and 0.25. Its diurnal pattern followed a decline during the morning hours, followed by an increase later in the day. At 8:00 and 20:00, when light intensity was relatively low, there were no significant differences in Y_(NO)_ among treatments, and in some cases, the values under 40- and 50-day drought stress were even significantly higher than those under 10-, 20-, and 30-day drought stress. However, during periods of high light intensity (12:00, 14:00, 16:00, and 18:00), Y_(NO)_ significantly decreased as drought duration increased. These results indicate that *C. caput-medusae* seedlings adapt to varying light intensities and drought stress levels by modulating Y_(II)_, Y_(NPQ)_ and Y_(NO)_. In terms of light energy utilization, the absorbed energy progressively shifted from photochemical use Y_(II)_ to Y_(NPQ)_ with increasing drought severity (**Table 2**).

## 4 Discussion

### 4.1 Response of chlorophyll fluorescence parameters to drought stress

Plant growth status serves as an integrative measure of a plant’s adaptability to environmental conditions (Jaffar et al., 2024a). Parameters such as plant height, basal stem diameter and crown width are important indicators of plant growth, and their relative increments reflect the developmental stage and adaptive responses of plants to environmental changes (Guo et al., 2014; Jaffar et al., 2024b). Under drought stress, plant height, basal stem diameter and crown width of *C. caput-medusae* seedlings initially increased and subsequently decreased with prolonged drought duration, indicating short-term or moderate drought may stimulate growth responses. This pattern indicates a potential adaptive mechanism in which seedlings allocate resources toward shoot development under mild water stress. However, as drought persists or intensifies, growth is inhibited. These findings imply that moderate drought stress might temporarily enhance seedling growth, although the physiological mechanisms driving this response such as osmotic adjustment or hormonal regulation require further investigation through targeted studies.

Chlorophyll fluorescence is closely associated with the various processes involved in photosynthesis (Jaffar et al., 2023). The effects of stress conditions on each stage of plant photosynthesis can be reflected through the kinetic changes induced by intrinsic chlorophyll fluorescence (Hájková et al., 2019). As such chlorophyll fluorescence parameters provide valuable insights into the physiological characteristics and stress responses of native plants (Xue et al., 2023).

In the later stages of drought stress, atmospheric humidity exhibited a narrow range of variation. PAR reflects the intensity of light energy available for plant absorption; LEF reflects the efficiency of electron transfer during photosynthesis (Lu et al., 2017); and NPQ reflects the proportion of excess light energy dissipated as heat by the PSII reaction center, serving as a protective mechanism against photodamage (Guan and Gu, 2009). In this study, PAR was significantly positively correlated with LEF and NPQ in *C. caput-medusae* seedlings under different drought stress treatments, and significantly negatively correlated with Y(II). These results suggest that *C. caput-medusae* seedlings can regulate and dissipate excess light energy through enhanced NPQ, thereby protecting the photosynthetic apparatus under drought stress. Similarly, Wei et al. (2021) reported that increase NPQ improves plant stress resistance by dissipating excess energy of PSII. Under the severe drought stress, elevated NPQ levels are likely to contribute to photoprotection by promoting thermal energy.

The energy dissipation of PSII following light absorption is partitioned into three components: actual photochemical efficiency (Y_(II)_), regulatory energy dissipation (Y_(NPQ)_) and non-regulatory energy dissipation (Y_(NO)_) (Kramer et al., 2004; Zhang et al., 2018; Itam et al., 2024). Y_(II)_ represents the effective photochemical efficiency of *C. caput-medusae* seedlings, while Y_(NPQ)_ denotes the quantum yield that regulated energy dissipation at PS II, serving as an important indicator of photoprotective activity. A high Y_(NPQ)_ indicates that plants are effectively dissipating excess energy to protect the photosynthetic apparatus. In contrast, a low Y_(NPQ)_ coupled with elevated (Y_(NO)_) reflects passive energy dissipation and fluorescence emission, indicating that the photochemical energy conversion and regulatory mechanisms are insufficient to fully dissipate absorbed light energy. This condition implies that the incident light intensity surpasses the plant’s capacity for energy utilization and regulation.

Under the drought stress test, LEF of *C. caput-medusae* seedlings initially increased and then decreased with increasing drought intensity, a pattern that paralleled changes in PAR, which also increased firstly and then decreased during the drought stress period. As drought intensified, the electron transport rate in the seedlings declined (Zhang et al., 2018), while the NPQ of seedlings increased. This indicates that with the prolonged drought stress, *C. caput-medusae* dissipated excess light energy as heat to protect the integrity of its photosynthetic apparatus. Similar responses have been reported by Itam et al. (2024) who revealed the increased NPQ in perennial ryegrass and Kentucky bluegrass under early drought stress conditions, followed by a decrease under prolonged stress. Zait et al. (2024) also found elevated NPQ in four *Cassia* plants species under drought stress. These findings are consistent with our results, confirming that persistent drought stress leads to an increase in NPQ. The enhanced thermal dissipation helps *C. caput-medusae* seedlings mitigate photoinhibition and photooxidation caused by excess excitation pressure on PSII.

### 4.2 The adaptation mechanisms of plants to drought stress

With the persistence of drought stress, Y_(II)_ of *C. caput-medusae* seedlings showed a gradual decline, indicating a reduction in the photochemical conversion capacity of PSII. This suggests that drought stress negatively impacts the efficiency of photosynthetic energy conversion. Similar findings were reported by Maia et al. (2020), who observed a decrease in the actual photochemical efficiency of PSII in sugarcane with increasing drought duration. In the present study, seedlings appeared to mitigate drought-induced stress over a 50-day period by activating self-regulatory mechanisms that rapidly dissipated excess light energy and protected the photosynthetic apparatus. This aligns with the findings of Ren et al. (2015), who also showed that plants enhance non-radiative heat dissipation under stress conditions to maintain the functional integrity of the photosynthetic system. The non-regulatory energy dissipation Y_(NO)_ showed minimal fluctuation during the whole drought stress period, and a significant difference between Y_(NO)_ was observed after 50 days of stress compared to other treatments. In summary, *C. caput-medusae* seedlings adapt to drought stress environment through protecting their photosynthetic system machinery through regulated energy dissipation mechanisms.

## 5 Conclusion

Drought is one of key limiting factors affecting plant survival in extremely arid desert environments. This study demonstrated that *C. caput-medusae* seedlings adapt to drought stress through modulating chlorophyll fluorescence parameters. Drought reduced both the actual photochemical efficiency and electron transport rate, while excess light energy was dissipated through enhanced thermal dissipation and other protective mechanisms to safeguard the photosynthetic apparatus. During the first 30 days of drought stress, PSII reaction centers remained functionally stable, with increased capacity for electron transport and heat dissipation. However, prolonged drought stress beyond 30 days elevated the risk of photoinhibition, indicating potential vulnerability of the photosynthetic system to extended water deficit. These findings improve the understanding of the drought adaptation strategies of *C. caput-medusae* and provide a theoretical basis for its sustainable use in shelterbelt construction in hyper-arid regions. It is important to note that this study was limited to *C. caput-medusae* seedlings; therefore, future studies should examine long-term drought adaptation mechanisms in mature individuals and across multiple *Calligonum* species to provide more ecologically relevant insights for vegetation restoration in arid environments.

## Data Availability

The original contributions presented in the study are included in the article/supplementary material. Further inquiries can be directed to the corresponding authors.
